# Chronic Training Induces Metabolic and Proteomic Response in Male and Female Basketball Players: Salivary Modifications during In-Season Training Programs

**DOI:** 10.3390/healthcare11020241

**Published:** 2023-01-12

**Authors:** Simone Luti, Rosamaria Militello, Gabriella Pinto, Anna Illiano, Angela Amoresano, Giovanni Chiappetta, Riccardo Marzocchini, Pietro Amedeo Modesti, Simone Pratesi, Luigia Pazzagli, Alessandra Modesti, Tania Gamberi

**Affiliations:** 1 Department of Biomedical, Experimental and Clinical Sciences “Mario Serio”, University of Florence, Viale G. Morgagni, 50, 50134 Florence, Italy; 2 Istituto Nazionale Biostrutture e Biosistemi, Viale delle Medaglie d’Oro, 305, 00136 Rome, Italy; 3 Department of Chemical Sciences, Polytechnic and Basic Sciences School, University of Naples Federico II, Via Cintia, 21, 80126 Napoles, Italy; 4 Biological Mass Spectrometry and Proteomics Group, SMBP, PDC CNRS UMR, 8249, ESPCI Paris, Université PSL, 10 rue Vauquelin, 75005 Paris, France; 5 Department of Experimental and Clinical Medicine, University of Florence, Largo Brambilla, 3, 50134 Florence, Italy; 6 Sport Medicine Unit, Careggi University Hospital, University of Florence, Largo Brambilla 3, 50134 Florence, Italy

**Keywords:** physical exercise, basketball, saliva, metabolomics, proteomics, training, sport, sex

## Abstract

The aim of this study was to characterize the salivary proteome and metabolome of highly trained female and male young basketball players, highlighting common and different traits. A total of 20 male and female basketball players (10 female and 10 male) and 20 sedentary control subjects (10 female and 10 male) were included in the study. The athletes exercised at least five times per week for 2 h per day. Saliva samples were collected mid-season, between 9:00 and 11:00 a.m. and away from sport competition. The proteome and metabolome were analyzed by using 2DE and GC–MS techniques, respectively. A computerized 2DE gel image analysis revealed 43 spots that varied in intensity among groups. Between these spots, 10 (23.2%) were differentially expressed among male athletes and controls, 22 (51.2%) between female basketball players and controls, 11 spots (25.6%) between male and female athletes, and 13 spots (30.2%) between male and female controls. Among the proteins identified were Immunoglobulin, Alpha-Amylase, and Dermcidin, which are inflammation-related proteins. In addition, several amino acids, such as glutamic acid, lysine, ornithine, glycine, tyrosine, threonine, and valine, were increased in trained athletes. In this study, we highlight that saliva is a useful biofluid to assess athlete performance and confirm that the adaptation of men and women to exercise has some common features, but also some different sex-specific behaviors, including differential amino acid utilization and expression of inflammation-related proteins, which need to be further investigated. Moreover, in the future, it will be interesting to examine the influence of sport-type on these differences.

## 1. Introduction

Exercise is an important regulator of cellular metabolic pathways, and several studies have focused on blood sampling to measure metabolites to investigate adaptations and responses to acute and chronic exercise [1]. Acute and chronic training are two very different exercise paradigms. Chronic exercise or exercise training is a repeated number of exercise sessions over a short or long-term period, while acute is defined as a single session of exercise [2,3,4]. However, it is known that acute exercise, if continued, can lead to muscle tissue damage, releasing proteins such as creatine kinase, myoglobin, or troponin into the plasma, which can reach the saliva through active or passive flow [5]. Recently, Franco-Martínez et al. [6] found sex-specific differences between men and women in the salivary proteome at rest and after acute exercise and concluded that sex is the factor that most strongly modulates salivary protein content. For these reasons, saliva is a useful alternative to blood to study the molecules involved in exercise adaptation [7].

The measurement of total salivary content may provide a non-invasive, low-stress method for assessing exercise-associated metabolic, hormonal, and immunologic status and for evaluating exercise load while avoiding upper-respiratory-tract infections. Recently, many studies have used saliva to assess concentrations of these compounds in response to exercise and training [8], and its composition reflects systemic health status [9]. Saliva is a hypotonic fluid composed mainly of water; electrolytes; and biomolecules such as proteins [10], cellular and bronchoalveolar debris, nasal secretions [11], and secretions from the salivary glands, which are composed of serous and mucous cells (acinar cells) and different types of duct cells, and these components contribute differently to the composition of saliva [12]. However, the major limitation in salivary samples is the inter-individual variability, which is determined by various factors, such as sex, age, and circadian rhythms, that may affect salivary composition, thus making a comparison between patients difficult [13,14].

Despite these limitations, it is interesting to monitor the concentration changes of salivary metabolites that occur during strenuous exercise. Pitti et al. [15] reported an altered concentration of 56 metabolites after a football match in the saliva of female athletes. They concluded that these modifications could be due to changes in water content rather than metabolite synthesis, especially after intense effort. In our previous studies, to delve deeper into sex-specific adaptations to chronic in-season training, we examined oxidative status, adiponectin, cortisol, steroid hormones, and fatty acid levels in the plasma of young male and female athletes compared to matched sedentary controls [16,17]. We concluded that the skeletal muscles of female athletes who train regularly during the competitive season have higher plasma concentrations of urea than those of their male counterparts, indicating increased protein catabolism that may lead to fatigue and overtraining. Females and males have different muscle properties, such as a different size, number, and diameter of muscle fibers, and this mainly explains the differences in maximum strength. This partly explains the sex differences in fatigability [18].

The aim of the present study is to investigate the sex-specific changes in the salivary proteome and metabolome of female and male basketball players during chronic training and away from competitions. Since many studies have already been conducted on the salivary proteome and metabolome in response to acute exercise, our first objective was to determine the effects during continuous chronic training [19]. We also wanted to investigate the influence of sex in sport adaptations, since male and female players underwent the same type of training. Interestingly, in a previous study we analyzed the plasma proteome and metabolome of the same highly trained athletes [16,17]. Overall, we hypothesized that the salivary proteome and metabolome may reflect the sex-specific sport adaptation identified in plasma samples. Indeed, in plasma, we found that regular exercise reduced the concentrations of proteins involved in chronic inflammation in females [17]; similarly, in this study, we found lower concentrations of inflammation-related proteins, such as Immunoglobulin A, Alpha-Amylase, and Dermcidin, in female athletes, compared to male players. 

## 2. Materials and Methods

Unless specified, all reagents were obtained from Bio-Rad Laboratories (Hercules, CA, USA). Solvents used for the sample preparation and LC–MS/MS analysis have >99.9% purity, as reported by the manufacturing companies. Acetonitrile (ACN) CHROMASOLV was obtained from Honeywell (Charlotte, NC, USA). Methanol (MeOH) and formic acid (HCOOH) were purchased from Sigma (St. Louis, MO, USA).

### 2.1. Participants

Students from the University of Florence enrolled in a degree course in Motor Sciences, Sport, and Health were recruited as sedentary subjects, while basketball players from local sports clubs, “US AFFRICO Firenze”, were selected. All participants were adults of Caucasian origin aged between 18 and 30 years. Women were randomly selected in relation to menstrual cycle. All subjects completed a physical activity questionnaire to determine their eligibility, using, in particular, the International Physical Activity Questionnaires (IPAQs) [20].

All participants were selected based on their health status, age, and stable body weight. Individuals who smoke and use antioxidants or dietary supplements were excluded from the study. All players attended the training sessions throughout the season, from September to May, and samples were collected during the 12th week. Athletes trained at least 5 times per week, with sessions of 2 h per day, as previously reported [17]. Briefly, the training protocol included both technical and aerobic exercises: 30 min of low–moderate running, followed by interval training runs to improve speed and simulate different game situations, 3 sets of 15 repetitions of exercises for the abdominals (oblique and the rectus abdominis muscles) and 3 sets of 12 repetitions of exercises for the shoulder muscles. Both male and female athletes followed the same training protocol [16].

Written informed consent was obtained from the participants; the research was carried out according to the policy statement set forth in the Declaration of Helsinki of 2013 and authorized by the local Ethics Committee of the University of Florence, Italy (AM_Gsport 15840/CAM_BIO). 

### 2.2. Saliva Samples Collection and Preparation

Subjects completed a questionnaire about their lifestyle habits and other general information. Saliva samples (3 mL) were collected in the morning, between 9:00 and 11:00 a.m., to avoid fluctuations due to circadian rhythms, before daily training sessions and outside of competition. Participants were instructed not to eat, drink, or brush their teeth for 30 min prior to collection. Saliva samples from each participant were collected by using Salivette Cortisol (Sarstedt AG and Co., Nümbrecht, Germany) and were stored in a freezer at −80 °C until their use. The cotton sliver of the Salivette was taken out and placed in the sublingual for 1/2 min and then put back into the Salivette. Samples were then centrifuged at 2000 rpm for 2 min. Saliva samples were grouped in four different pools, i.e., one for each group involved in the study; therefore, each pool consisted of the saliva samples of 10 subjects. For proteomic analysis, saliva pools were precipitated overnight at −20 °C with ice-cold acetone (1:5) and then centrifuged for 30 min at 9000 rpm. The acetone was then removed, and the residuals were left to evaporate. A total of 150 μL of rehydration solution composed of 8 M urea, 4% (*w*/*v*) 3-((3-cholamidopropyl) dimethylammonio)-1-propanesulfonate (CHAPS), and 50 mM Dithiothreitol (DTT) was used to suspend the pellets. A Bradford assay was carried out to assess the total protein contents.

### 2.3. Two-Dimensional Polyacrylamide Gel Electrophoresis (2DE)

A total of 30 µg of protein samples was loaded on 11 cm IPG strips, pH 3–10 NL (Bio-Rad Laboratories, Hercules, CA, USA), that were actively rehydrated for 16 h (at 50 V) in 200 µL of rehydration solution supplemented with 0.5% (*v*/*v*) carrier ampholyte (Bio-Rad Laboratories, CA, USA) and a trace of bromophenol blue. Isoelectric focusing (IEF) was carried as follow: 250 V for 20 min (rapid), from 250 to 8000 V for 1 h, and then 8000 V until a total of 23,000 V/h was reached, with a limiting current of 50 µA/strip. Strips were then equilibrated for 15 min in 6M urea, 30% (*v*/*v*) glycerol, 2% (*w*/*v*) SDS, and 2% (*w*/*v*) DTT in 0.05 M Tris-HCl buffer (pH 6.8), and for 15 min in the same solution with 2.5% (*w*/*v*) iodoacetamide instead of DTT. The strips were moved on 9–16% polyacrylamide linear gradient gels, and SDS–PAGE was performed at 200 V until the dye front reached the bottom of the gel. Ammoniacal silver nitrate stain was used for analytical gels, as previously described [21]. For preparative gel, 200 µg of proteins (50 µg from each pool) was used, and the gel was stained with colloidal Coomassie blue G-250 [22].

### 2.4. Protein Identification by Mass Spectrometry

#### 2.4.1. In Situ Digestion of 2DE Spots

Each 2DE spot was manually picked up from Coomassie-stained preparative gels and subjected to in situ digestion, as previously described [17]. Peptide mixtures were eluted from each gel and recovered after several washings by using 0.1% formic acid (HCOOH) and acetonitrile (ACN). Finally, the peptide mixture was vacuum-dried and resuspended in 2% ACN containing 0.1% HCOOH for the subsequent liquid chromatography–tandem mass spectrometry (LC–MS/MS) analysis. 

#### 2.4.2. LC–MS/MS Analysis

A 6520 Accurate-Mass Q-TOF LC/MS system (Agilent Technologies, Santa Clara, CA, USA) equipped with a 1200 HPLC system and a chip cube (Agilent Technologies, Santa Clara, CA, USA) was used to analyze the peptide mixtures. The peptide mixture (1 µL) was automatically injected by an autosampler and desalted at a flow rate of 4 µL/min in a 40 nL enrichment column with 0.1% HCOOH as an eluent. A C18 reverse-phase capillary column (75 mm × 43 mm) included into an Agilent Technologies chip (Santa Clara, CA, USA) was used to fractionate the sample at a flow rate of 400 nL/min, with a linear gradient of eluent B (0.1% HCOOH in 95% ACN) in A (0.1% HCOOH in 2% ACN) from 5 to 80% in 50 min.

An analysis of the peptide was carried out by using the data-dependent acquisition of one MS scan (mass range *m*/*z* 300–2400), followed by an MS/MS scan of the five most abundant ions in each MS scan. MS/MS spectra were measured automatically when the MS signal was greater than the threshold of 50,000 counts. Charge ions preferably isolated were double, triple, and quadruple, and they were fragmented over singly charged ions. The acquired MS and MS/MS data were converted in a .mgf file, using Mass Hunter software (Agilent Technologies, Santa Clara, CA, USA), in order to be used for protein identification, with a licensed version of Mascot Software (London, UK). Searches were performed by using UniProtKB and the NCBI protein database with *Homo sapiens* taxonomy. Mascot search parameters were as follows: trypsin as an enzyme, allowed number of missed cleavage 3; carbamidomethyl, C as fixed modifications; oxidation of methionine (Met), pyro-Glu (N-terminal Gln and Glu) as variable modifications and peptide charge from +2 to +4. A mass tolerance value of 10 ppm was set for the precursor, and 0.2 was set for the fragment ions. Proteins identified with at least 1 peptide displaying a *p*-value < 0.05 were selected as significant.

### 2.5. Western Blot Analysis

An equal amount of four different pools of saliva samples (10 µg of total proteins) was combined with 4× Laemmli buffer (0.5 M TrisHCl pH 6.8, 10% sodium dodecyl sulfate (SDS), 20% glycerol, β-mercaptoethanol, and 0.1% bromophenol blue) and boiled for 5 min. After that, 12% SDS/ polyacrylamide gel was used to separate samples that ware then transferred onto polyvinylidene fluoride membrane (PVDF), using the Trans-Blot Turbo Transfer System (BIO-RAD Laboratories, CA, USA). The PVDF was probed with primary antibody Alpha-Amylase (sc-46657), Immunoglobulin A (IgA) (sc-166334), and Dermcidin (sc-33656), all provided by Santa Cruz Biotechnology (Santa Cruz Biotechnology, Santa Cruz, CA, USA), and Cystatin A (GT2264), which was provided by GeneTex (GeneTex, Irvine, CA, USA); diluted 1:1000 in 2% milk; and incubated overnight at 4 °C. An enhanced chemiluminescence (ECL) detection system (GE Healthcare, Chicago, IL, USA) and Amersham Imager 600 (GE Healthcare, IL, USA) were used to detect the bands after incubation with horseradish peroxidase (HRP)-conjugated anti-mouse IgG (1:10,000) (Santa Cruz Laboratories, Santa Cruz, CA, USA). The PVDF membrane was stained with Coomassie brilliant blue R-250 blot, and the total protein intensities were used to normalize the intensity of the immuno-stained bands. The blot was analyzed by using the ImageJ program, as described previously [23]. 

### 2.6. Analysis of Salivary Samples by Gas Chromatography–Mass Spectrometry (GC–MS) 

Metabolite analyses were performed on four pools of salivary samples, one for each group (BM, BF, CM, and CF). The same volume of saliva from each participant was taken to make each pool in order to reduce intraindividual variability of metabolites levels. The GC–MS analysis was carried out on 150 µL of saliva, as reported by Luti et al. 2020 [18], but with slight modifications. Briefly, 150 µL of the samples was mixed with 150 μL ice-cold MeOH (#34854-1; Sigma Aldrich, Darmstadt, Germany) containing 20 µM norvaline (#N7502; Sigma Aldrich), which served as an internal standard, and 150 μL of chloroform (ratio 1:1:1; #900688-1; Sigma Aldrich). Then samples were vortexed at 4 °C for 20 min, centrifuged at 8000× *g* for 10 min at 4 °C, and the upper phase was collected in a new tube and evaporated at room temperature in a rotavapor. 

For derivatization, dried polar metabolites were dissolved in 30 µL of 2% methoxyamine hydrochloride (#226904; Sigma Aldrich) in pyridine (25104; Thermo Fisher Scientific, Waltham, MA, USA) and held at 30 °C for 90 min. After dissolution and reaction, 45 mL MSTFA + 1% TMCS (69478-10x; Sigma Aldrich) was added, and samples were incubated at 37 °C for 60 min. Gas chromatographic runs were performed with helium as carrier gas at 0.6 mL/min at an inlet temperature of 250 °C. The GC oven temperature ramp was from 60 to 325 °C, at 10 °C/min. The data acquisition rate was 10 Hz. For the Quadrupole, an EI source (70 eV) was used, and full-scan spectra (mass range from 50 to 600) were recorded in the positive ion mode. The injection volume was 1 µL, and a split ratio of 1:10 was used. 

Global metabolic profiling was obtained by using a MassHunter data-processing tool (Agilent). Metabolite identification was performed at level 1, as proposed by the metabolomics standards initiative [24], using a retention index and mass spectrum as the two independent and orthogonal data required for identification. A Fiehn Metabolomics RTL library (Agilent G1676AA) was used as the reference library of compounds [25]. For the identification of significant metabolic pathways involved in male and female in-season chronic training, we used MetaboAnalyst 4.0 (http://www.metaboanalyst.ca, date: 10 April 2022). A *p*-value < 0.05 and a false discovery rate (FDR) < 0.1 were used to select the involved pathways [26].

### 2.7. Image and Statistical Analysis

Data are presented as means +/− standard deviation (SD) from at least three experiments. Amersham Imager 600 (GE Healthcare) was used to scan the 2DE images which were saved with a resolution of 300 dpi and in 16-bit TIFF format. Progenesis SameSpots software v4.0 (Nonlinear Dynamics, UK) was used to perform image analysis after spots detection and background subtraction. We selected as the reference image the gel scan showing the best protein pattern, and its spots were used to match, quantify, and normalize the spots volume across all the other gels. The default parameters of the Progenesis SameSpots Stat module were used to perform the statistical analysis and principal component analysis. The differentially expressed spots were identified by using one-way ANOVA (*p*-value < 0.05), and to find out the significant differences between groups, Tukey’s multiple comparisons test, using GraphPad Prism v8.0 software, was performed. The Tukey test compared every mean with every other mean, computing a confidence interval for multiple comparisons of 95% confidence. Significance was defined as *p*-value < 0.05.

We checked the normality and homogeneity distribution of our data by the D’Agostino–Pearson and Brown–Forsythe tests, respectively. As a measure of the effect size, we reported the eta-squared (η^2^) calculated in ANOVA (Tukey’s multiple comparisons test) performed on all groups together, using GraphPad Prism 8, considering the following intervals: 0.01–0.20 = small effect, 0.21–0.60 = moderate effect, and 0.61–0.99 = high effect [16]. The power analysis performed post hoc with GPower 3.1 confirmed that the sample size is satisfactory for a power of 0.90 and *p* < 0.05 for each variable analyzed, with the only exception being L-glutamic acid, for which the power is 0.5.

## 3. Results

The characteristics of 20 professional basketball players (10 female and 10 male) and 20 sedentary participants (10 male and 10 female) as controls are reported in Table 1.

In summary, the mean age of all participants was 24.4 ± 4.2 years, and no significant differences were found in the Body Mass Index (BMI) between the player groups and the control groups (*p*-value = 0.64 for males, and *p*-value = 1 for females; η^2^ = 0.084) and between males and females (*p*-value = 0.18 for athletes, and *p*-value = 0.72 for controls; η^2^ = 0.084), despite the fact that males were taller and heavier than females in both groups (athletes and controls) and female athletes were taller than the respective control. Moreover, all the professional basketball players, both male and female, trained at least five times a week, according to the specific training programs reported previously [16].

### 3.1. Overview of Saliva Protein Profiles

**Figure 1:**
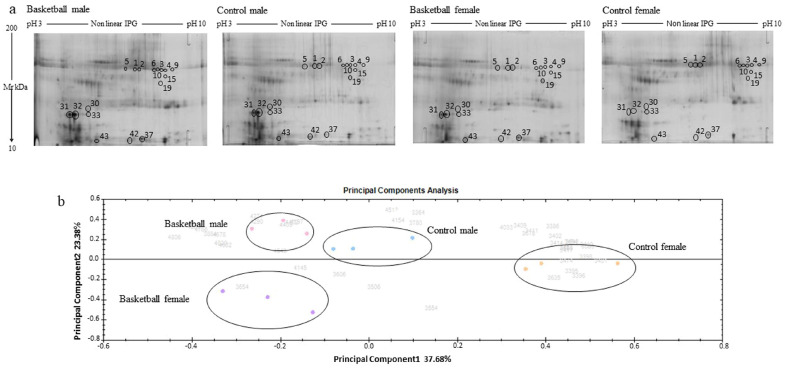
Proteomic profile of Basketball players and controls. (**a**) Representative 2DE images of silver-stained gels of saliva proteins run on NL pH 3–10 IP strip and in 9–16% polyacrylamide linear gradient. Circles and numbers indicate statistically differentially abundant proteins between the four groups analyzed, as reported in Table 2. (**b**) Multivariate analysis of the 2DE gel images results using Principal Components Analysis (PCA) performed by Progenesis SameSpots 4.0 software (Nonlinear Dynamic, UK).

The 2DE silver-stained gel images were analyzed by Progenesis SameSpots software 4.0 (Nonlinear Dynamics, UK), using default parameters. A representative gel for each group was reported in Figure 1a. Each saliva sample was run in triplicate to obtain statistically significant results. After automatic spot identification, an average of about 1157 protein spots was detected in each gel. The computational 2DE gel image analysis pointed out 72 differentially expressed protein spots (ANOVA *p*-value < 0.05), while the Tukey’s test showed that 43 of them were differentially expressed in groups that can be compared to each other, as reported in Supplementary Table S1: the terms of comparison chosen were the practice of sport and sex. Among these spots, 10 (23.2%) were differentially expressed between male athletes and controls, 22 (51.2%) between female basketball players and controls, 11 spots (25.6%) were differentially expressed between male and female athletes, and 13 spots (30.2%) between male and female controls.

### 3.2. Principal Component Analysis

A multivariate analysis PCA (principal component analysis) was carried out to obtain an overview of the proteomic data for overall trends in all groups. In the PCA biplot shown in Figure 1b, each dot describes the collective expression profiles of one sample; gels were grouped according to the difference of protein spot abundance, and the plot demonstrates consistent reproducibility among repeated samples within each group. The PCA biplot reveals four distinct main protein profile groups corresponding to the (i) basketball male group (pink circle), (ii) basketball female group (violet circle), (iii) control male group (blue circle), and (iv) control female group (orange circle). The first principal component, which distinguished 37.68% of the variance, clearly separates the proteome data of female controls group from the other groups, while the second component, with an additional 23.38% of variance, clearly distinguished the basketball male group from the basketball female group. The PCA plot suggests that sport training drastically affects the protein pattern; in fact, the greater differences are evident between the athletes than between the controls. 

### 3.3. Proteins Differentially Modulated by Exercise and Sex

The proteins modulated by chronic in-season training or sex identified by the mass spectrometry approach are reported in Table 2. By comparing the proteins differentially expressed between athletes and sedentary controls, we found the protein Collagen-1(I) chain (P02452) to be downregulated in both male and female athletes (fold change −2.9 in male and −2.6 in female). We found three proteins, Immunoglobulin heavy constant alpha 1 and alpha 2 (P01876 and P01877, respectively) and the protein Alpha-Amylase 1A (P0DTE8) detected in multiple spots within the gel and with different fold changes, and it is likely that different forms of the same protein, processed or post-translationally modified, migrate differently. Post-translational modifications of Immunoglobulins are very frequent and often are associated with physiological and pathological conditions [27], while proteomic studies reported that Alpha-Amylase could be detected in more than twenty spots on 2DE gels. Indeed, posttranslational modifications can give rise to a pattern of isozymes [28,29].

**Table 2:** Differentially expressed protein spots from 2DGE identified by LC–MS/MS analysis.

Spot No. ^a^	AC ^b^	Gene Name	Protein Name	Score ^c^	Protein Mass	Protein Cover ^d^	Tukey’s Test ^e^/Fold Change ^f^			
							BM vs. CM	BF vs. CF	BM vs. BF	CM vs. CF
1	P0DTE8	AMY1A	Alpha-Amylase 1A	791	56,484	52.2	ns	*/−2.6	ns	ns
	P01876	IGHA1	Immunoglobulin heavy constant alpha 1	243	38,486	22.9	ns	*/−2.6	ns	ns
	P01877	IGHA2	Immunoglobulin heavy constant alpha 2	170	37,366	19	ns	*/−2.6	ns	ns
2	P0DTE8	AMY1A	Alpha-Amylase 1A	628	56,484	31.9	ns	*/−2.6	ns	ns
	P01876	IGHA1	Immunoglobulin heavy constant alpha	141	38,486	14.7	ns	*/−2.6	ns	ns
	P01877	IGHA2	Immunoglobulin heavy constant alpha 2	121	37,366	13.9	ns	*/−2.6	ns	ns
3	P0DTE8	AMY1A	Alpha-Amylase 1A	1120	56,484	52.8	*/−2.9	**/−2.6	ns	*/−1.7
	P02452	COL1A1	Collagen alpha-1(I) chain	117	37,077	11.3	*/−2.9	**/−2.6	ns	*/−1.7
	P01876	IGHA1	Immunoglobulin heavy constant alpha 1	223	38,486	20.2	*/−2.9	**/−2.6	ns	*/−1.7
	P01877	IGHA2	Immunoglobulin heavy constant alpha 2	176	37,366	16.2	*/−2.9	**/−2.6	ns	*/−1.7
4	P0DTE8	AMY1A	Alpha-Amylase 1A	1073	56,484	57.1	ns	**/−3.3	ns	ns
	P01876	IGHA1	Immunoglobulin heavy constant alpha 1	194	38,486	16.6	ns	**/−3.3	ns	ns
	P01877	IGHA2	Immunoglobulin heavy constant alpha 2	143	37,366	12.6	ns	**/−3.3	ns	ns
	A0N4V7	Tcr-alpha	putative T-cell receptor beta	39	2269	38.1	ns	**/−3.3	ns	ns
5	P0DTE8	AMY1A	Alpha-Amylase 1A	478	56,484	30	**/1.4	**/−1.5	****/1.9	ns
6	P0DTE8	AMY1A	Alpha-Amylase 1A	1011	56,484	48.8	ns	*/−2.4	ns	ns
	P01876	IGHA1	Immunoglobulin heavy constant alpha 1	254	38,486	25.7	ns	*/−2.4	ns	ns
	P01877	IGHA2	Immunoglobulin heavy constant alpha 2	212	37,366	21.9	ns	*/−2.4	ns	ns
9	P0DTE8	AMY1A	Alpha-Amylase 1A	904	58,415	43.2	ns	*/−3.2	ns	ns
	P68871	HBB	Hemoglobin subunit beta	147	16,102	35.4	ns	*/−3.2	ns	ns
	P01876	IGHA1	Immunoglobulin heavy constant alpha 1	240	38,486	21.5	ns	*/−3.2	ns	ns
	P01877	IGHA2	Immunoglobulin heavy constant alpha 2	179	37,366	16.2	ns	*/−3.2	ns	ns
10	P0DTE8	AMY1A	Alpha-Amylase 1A	911	58,415	41.1	*/−2.3	ns	ns	ns
	P01876	IGHA1	Immunoglobulin heavy constant alpha 1	211	38,486	17.3	*/−2.3	ns	ns	ns
	P01877	IGHA2	Immunoglobulin heavy constant alpha 2	175	37,366	16.2	*/−2.3	ns	ns	ns
15	P0DTE8	AMY1A	Alpha-Amylase 1A	375	58,415	18	ns	*/−2.7	ns	ns
	P25311	AZGP1	Zinc-alpha-2-glycoprotein	390	34,465	37.6	ns	*/−2.7	ns	ns
	Q01469	FABP5	Fatty acid–binding protein 5	147	15,497	28.1	ns	*/−2.7	ns	ns
19	P0DTE8	AMY1A	Alpha-Amylase 1A	831	58,415	44.2	ns	**/−2	ns	**/−2.2
30	P02768	ALB	Albumin	269	71,317	15.1	ns	ns	ns	*/2.1
	P07355	ANXA2	Annexin A2	249	38,808	20.6	ns	ns	ns	*/2.1
	P81605	DCD	Dermcidin	208	11,391	38.2	ns	ns	ns	*/2.1
31	P01040	CSTA	Cystatin A	246	11,000	98	ns	ns	ns	*/3.8
	P81605	DCD	Dermcidin	177	11,391	35.5	ns	ns	ns	*/3.8
32	P01040	CSTA	Cystatin A	348	11,000	94.9	ns	ns	ns	*/4.3
	P81605	DCD	Dermcidin	73	11,391	30.9	ns	ns	ns	*/4.3
33	P02768	ALB	Albumin	675	71,317	28.1	ns	ns	**/2.7	ns
	P81605	DCD	Dermcidin	150	11,391	30.9	ns	ns	**/2.7	ns
	P01876	IGHA1	Immunoglobulin heavy constant alpha 1	214	38,486	15.3	ns	ns	**/2.7	ns
42	P02768	ALB	Albumin	190	68,408	20.5	ns	ns	ns	*/2.1
	P31151	S100A7	Psoriasin	115	11,564	62.4	ns	ns	ns	*/2.1
43	P81605	DCD	Dermcidin	143	11,391	37.3	ns	ns	ns	*/3

^a^ Spot numbers reported in the representative 2DE images shown in Figure 1. ^b^ Accession number in Swiss-Prot/UniProtKB (http://www.uniprot.org/). ^c^ MASCOT MS score (Matrix Science, London, UK; http://www.matrixscience.com). MS matching score greater thank 56 was required for a significant MS hit (*p*-value < 0.05). ^d^ Sequence coverage = (number of the identified residues/total number of amino acid residues in the protein sequencer) × 100%. ^e^ Tukey’s post hoc test was performed on ANOVA *p*-values by GraphPad Prism 6.0 software (* *p*-value < 0.05), (** *p*-value < 0.01), (**** *p*-value < 0.0001), and (ns = not significant). ^f^ Fold change was calculated by GraphPad Prism 6.0 software. It is the ratio of the mean normalized spot volumes of male basket group (BM), male control group (CM), female basket group (BF), and female control group (CF). It was reported only for statistically significant values.

As reported above, we observed that exercise’s effects on saliva proteins were not similar for males and females. Moreover, we also evaluated the possible sex-related differences in sedentary controls. In the male controls, we found increased levels of the proteins Dermcidin (P81605) (Spots 30, 31, 32, and 43) and Cystatin-A (P01040), which were both found in more of one spot (Spots 31 and 32), and Albumin (P02768) (42). Dermcidin was also found in Spot 33, where its modulation is related to sport and sex, and it is upregulated in male athletes (fold change 2.7).

### 3.4. Saliva IgA, Alpha-Amylase, Dermcidin, and Cystatin a Determination

**Figure 2:**
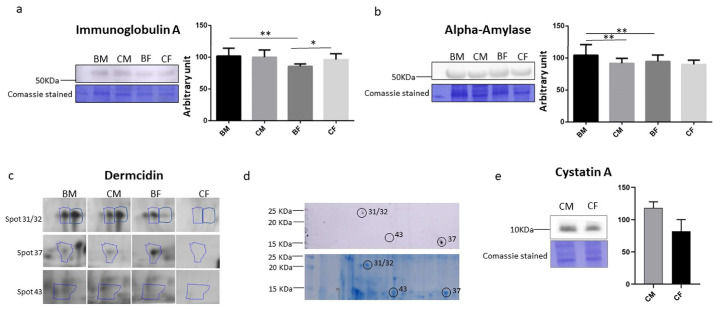
Validation of proteomic results. Histograms and representative immunoblot images of (**a**) Immunoglobulin A and (**b**) Alpha-Amylase in BM (basketball male group), CM (control male group), BF (basketball female group), and CF (control female group). Normalization of immunoblot was performed on Coomassie-stained PVDF membrane. The statistical analysis was carried out by two-tailed *t*-test, using Graphpad Prism 6 (* *p* < 0.05; ** *p* < 0.01). (**c**) Representative image of 2DE silver-stain spots, (**d**) 2DE Western blot for Dermcidin, and (**e**) Cystatin A.

To confirm the differential expression levels of the identified proteins, we performed a Western blot analysis of IgA and Alpha-Amylase, whose expression increased in male athletes compared with female players (Figure 2a,b). The Western blot analysis confirms the results obtained by the proteomic analyses; in fact, IgA was lower in female athletes in comparison to male players, as reported in Spot 33, and in comparison to control females, as reported in Spots 1, 2, 3, 4, and 6. For Alpha-Amylase, the Western blot analysis confirms the trend observed in Spot 5, in which only this protein was identified. However, it does not validate the decreased level in basketball female players in comparison to their controls observed also in other spots, such as Spots 1, 2, 3, 4, 6, 9, and 15. It is important to underline that, in all the spots, more than one protein was identified, and the fold changes reported in Table 2 were calculated through an analysis performed on silver stain gel. The presence of different proteins in the same spot could explain why these results were not confirmed by the Western blot. Furthermore, IgA and Alpha-Amylase are abundant proteins in saliva, and, like albumin in blood, they appear over a wide range of pH values and molecular weights, they so were found in different spot interfering with proteomic studies. 

Dermcidin was found to be increased in the control males compared to the females in more than one spot (Spots 30, 31, 32, and 43; Figure 2c), so we decided to perform a Western blot analysis on 2DE gel, as reported in Figure 2d. We confirmed the presence of Dermcidin in Spots 31, 32, and 43, and we also identified Dermcidin in Spot 37 (Figure 2d) that was further validated by mass spectrometry, confirming the presence of this protein as reported in Table 3. In addition, the identification of Spot 43 shows Dermcidin as a unique protein, but its expression is modified only in the controls (increased in male vs. female). Spots 37 and 43 show a similar size but different isoelectric points, as is evident from the gel in Figure 1a. Dermcidin is secreted into sweat, where it is proteolytically processed and also post-translationally modified in order to give rise to antimicrobial peptides, thus explaining its presences in several spots [30]. Furthermore, it is also released by skeletal muscles, and the full-length protein stimulates apoptosis under hypoxic conditions [31].

Moreover, in Spots 31 and 32, another protein, Cystatin A, was identified with a higher protein coverage and score. In Figure 2e, the immunoblot quantifications were reported, confirming the results of Cystatin A modulation. 

### 3.5. Metabolites Differentially Modulated by Exercise and by Sex

**Figure 3:**
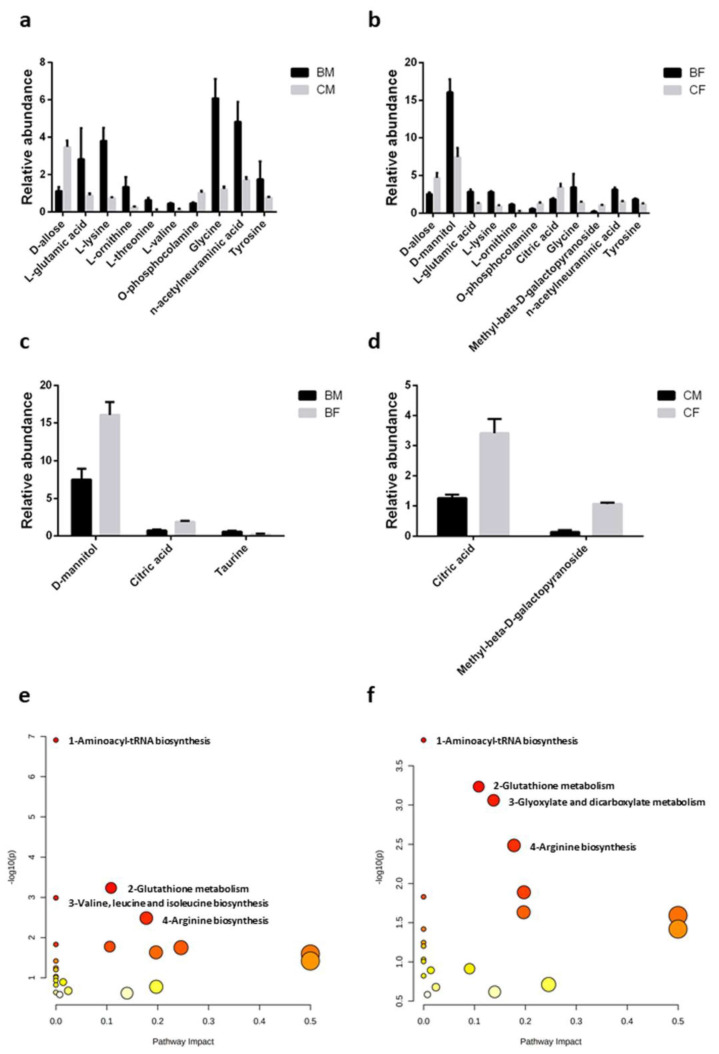
Plasma metabolomic profile of female and male basketball players. Histogram representation of saliva metabolites whose relative abundance is statistically different (*p* < 0.05) between (**a**) male basketball athletes and controls; (**b**) female basketball athletes and controls; (**c**) female and male basketball athletes; and (**d**) female and male controls (CM, control male group; BF, basketball female group; and CF, control female group). Statistical analysis was performed by two-way ANOVA (Tukey’s multiple comparisons test), using GraphPad Prism 6. Representation of the metabolic pathways involved in (**e**) male and (**f**) female in-season chronic training, using the differentially abundant metabolites reported in Table 4. For analysis, we used MetaboAnalyst 4.0, setting a *p*-value < 0.05 and a false discovery rate (FDR) < 0.1 to select the involved pathways.

The metabolic modulation of salivary samples due to the chronic training was evaluated through GC–MS, and each compound obtained was identified by the Fiehn library, which allows users to identify several salivary compounds differentially expressed between the groups (Figure 3 and Table 4). The overall identified metabolites were reported in Supplementary Table S2. Metabolite concentrations were normalized by using both saliva volume (for each group, 150 µL) and total metabolites concentration according to Pitti et al. [15] with similar results.

Comparing both male and female athletes with their respective sedentary controls, we identified several amino acids modulated by training and, more precisely, showing a statistically significant increase: L-glutamic acid (η^2^ = 0.3589), L-lysine (η^2^ = 0.8801), L-ornithine (η^2^ = 0.8508), glycine (η^2^ = 0.8007), and tyrosine (η^2^ = 0.7897). In addition to these, in male basketball players, two more amino acids, L-threonine (η^2^ = 0.8120) and L-valine (η^2^ = 0.5985), showed a significant increase compared to the sedentary controls. Again, when comparing both male and female athletes with the controls, we found a significant increase in the N-acetylneuraminic acid salivary level (η^2^ = 0.7202). On the contrary, other metabolites, such as D-allose (η^2^ = 0.8869) and O-phosphocolamine (η^2^ = 0.9167), decreased (Figure 3a,b and Table 4).

As with proteins, we observed that some metabolites were sex-specific during chronic exercise, suggesting a possible difference between the male and female athletes and the controls. When comparing male and female athletes, in the males, we found a significant decrease in D-mannitol (η^2^ = 0.8319) and citric acid (η^2^ = 0.9044) and a great increase in taurine (η^2^ = 0.7411) (Figure 3c; Table 4). With regard to citric acid, it is interesting to note that in sedentary controls, the increases are significant in females compared to males. Moreover, male controls showed a reduced value of methyl-beta-D-galactopyranoside (η^2^ = 0.9632) compared to the female controls (Figure 3d and Table 4).

We used metabolites showing significant modifications (Table 4) to identify the metabolic pathway related to chronic training and sex. These analyses were conducted by using MetaboAnalyst 4.0. From a total of 24 pathways indicated comparing athletes versus sedentary controls, 4 for male and 4 for female athletes were selected by following the criteria of *p* < 0.05 and FDR < 0.1 (Figure 3e,f). From all observed significant pathways, three of them were involved in both males and females. These pathways are as follows:

(i) Aminoacyl-tRNA biosynthesis—in detail, for males, glycine, L-valine, L-lysine, L-threonine, L-tyrosine, and L-glutamate (number 1 in Figure 3e), and for females, glycine, L-lysine, L-tyrosine, and L-glutamate (number 1 in Figure 3f);
(ii) Glutathione metabolism—for both males and females, glycine, L-glutamate, and L-ornithine (number 2 in Figure 3e,f)
(iii) Arginine biosynthesis; for both males and females, L-glutamate and L-ornithine (number 4 in Figure 3e,f).

On the other hand, valine, leucine, and isoleucine biosynthesis were significant only in males (L-valine and L-threonine in pathway number 3 panel in Figure 3e), while glyoxylate and dicarboxylate metabolism were significant only in females (citric acid, glycine, and L-glutamate in pathway number 3 in Figure 3f).

## 4. Discussion

In the present study, we investigated the sex-specific changes in the salivary proteome and metabolome in highly trained athletes during continuous chronic training. Several studies have been conducted on the salivary proteome and metabolome in response to acute training; very little is known about the repeated number of sessions [6,19,32]. Overall, we found reduced levels of proteins involved in chronic inflammation, such as Immunoglobulin A, Alpha-Amylase, and Dermcidin, in females, thus confirming the anti-inflammatory effect of exercise training. In addition, we found a training effect on metabolism related to glutathione biosynthesis in both male and female players, whereas an increase in branched chain amino acids was observed only in male players.

In a recent paper, McKetney et al. [33] reported a multiomics analysis in the saliva of soldiers before, during, and after a military deployment in which a military attack was simulated to examine the acute and chronic exercise response. However, in their experimental model, a stress condition due to the period of the military mission is foreseen. 

This study focused on determining the effects of chronic training on the salivary proteome and metabolome during the season. Both male and female basketball athletes were evaluated to highlight possible gender differences. Among proteins, significant modulations were observed in molecules with antimicrobial activity involved in mucosal immunity, such as salivary IgA and Alpha-Amylase [7].

In our experimental model, decreased expression of salivary IgA was observed in female athletes, suggesting that highly trained athletes may experience an immunosuppressive state during in-season training periods (Figure 2a). The same evidence was previously obtained in plasma samples from the same subjects [17]: indeed, we showed a reduction of several plasma proteins associated with chronic inflammation, enhancing the anti-inflammatory effect of regular training in females. Several studies reported lower salivary IgA levels as an acute effect of exercise [34,35], while other studies found no change [36] or even an increase [7,37], suggesting a possible role of exercise in modulating this protein. In addition, the salivary IgA level may be helpful to detect excessive exercise load, which may determine the risk of respiratory infections in elite athletes. However, in our results, we found that all identified isoforms were less expressed in female athletes compared to their controls, suggesting that the effect is more pronounced in females than in males [38,39].

The same trend was observed in the modulation of salivary Alpha-Amylase between male and female athletes. The highlighted data may indicate that the intensity, workload, and duration of training during season must take into account the sex difference. Specifically, we observed a lower level of salivary Alpha-Amylase in female athletes (Figure 2b) [40]. These proteins are an indicator of sympathetic nervous system activity under exercise conditions, and it is observed to decrease in saliva from elite athletes after a heavy training season, indicating a non-functional overreaching [41,42]. This decrease is more pronounced in the athlete groups than in the control groups (Figure 2b). The main interest of investigators in the studying muscle adaptation has been changes in the levels of this enzyme after acute exercise, but there are few data on its modulation after chronic training. As with salivary IgA levels, it is suggested that the stress response to physical exercise can be assessed by monitoring salivary Alpha-Amylase, since the salivary levels of both enzymes are altered in response to sympathetic and parasympathetic nervous system activation after exercise [7] and are known to be higher in most competent and experienced male athletes than in females [42,43].

In our condition, training protocols for male and female athletes were identical. This altered female-specific salivary IgA and Alpha-Amylase response could be due to an inadequate training workload. Consequently, training that is not specifically planned and modulated for female athletes could affect female athletic performance and, at best, result in lower performance. In summary, we demonstrated a stress response of the body in female athletes to chronically inadequate training.

In our previous study, we found reduced salivary cortisol levels and absence of oxidative stress in both female and male basketball players under the same conditions [16]. Kivlighan et al. [42] observed an inverse relationship between salivary cortisol and Alpha-Amylase during competition in female athletes and not in male athletes. Indeed, salivary cortisol levels increased in response to acute exercise in females. Moreover, Cadegiani et al. [44] reported that high resting cortisol levels are associated with overtraining. Both cortisol and Alpha-Amylase can be signs of stress in response to exercise [40,45], but they cannot be considered equivalent because Alpha-Amylase is produced locally in the salivary glands and is therefore a more direct and sensitive marker of exercise-induced stress than cortisol, which is transported from blood to saliva [46,47].

Regarding Dermcidin, our results have shown that this protein is present in saliva and that it is modulated during training. Dermcidin is expressed as a precursor that can be proteolytically processed to obtain various antimicrobial peptides. The peptides produced provide a line of defense and are abundant in body fluids. Moreover, this protein is expressed in various cell types, but its function remains to be elucidated. Recently Esposito et al. [31] identified Dermcidin as a novel myokine that modulates cardiac myocytes and their function. The authors proposed that this protein is a signaling molecule released from skeletal muscles. We can suggest that this myokine may be a factor in inter-organ communication in male athletes with higher skeletal muscle mass compared to females, affecting nearby or distant organs and stimulating various mechanisms, including inflammatory processes that may lead to muscle fatigue.

We found a higher saliva concentration of Cystatin A in male subjects compared with female controls; Cystatin A is a protein belonging to the Cystatin family, a group of cysteine proteases produced mainly in the submandibular glands. Under our experimental conditions, this protein was not increased during chronic exercise, whereas several authors reported that acute exercise significantly increased its salivary levels. We conclude that the lack of modulation in Cystatin A observed during chronic exercise is related to the time elapsed after the end of acute exercise [48]. However, this protein family seems to be linked to an increase in oxidative stress, as reported by some authors [49], and this could be related to our results in which all the athletes analyzed, both male and female, had a low level of this type of stress. 

The metabolites identified as significantly associated with chronic training in both male and female athletes are related to amino acid biosynthesis and translation, as evidenced by modulation of the aminoacyl-tRNA biosynthesis pathway. Moreover, both male and female athletes exhibited metabolites related to glutathione biosynthesis, indicating an increase in antioxidant defenses [50]. Regarding threonine and ornithine matching in the glutathione biosynthesis pathway, the first is converted to pyruvate to produce glucose, and its salivary level could be due to a mechanism to maintain glycemia in athletes. Ornithine is involved in the urea cycle, and its increase could indicate a rise in the urea cycle to eliminate ammonia produced by amino acid degradation during chronic training [51]. In males, but not in females, branched chain amino acids have been associated with an increase in body mass, as reported by Rodriguez-Carmona et al. [52], and in our experimental conditions, we found a significant increase in L-valine and L-threonine in the saliva of male athletes only. As expected, in male athletes, a training-related metabolic pathway is associated with valine, leucine, and isoleucine biosynthesis, considering that branched chain amino acids are associated with an increase in skeletal muscle mass, which is related to sex-specific differences in whole body muscle mass [53,54,55].

In our previous manuscript [16], we found increased amino acid metabolism in the plasma of male athletes, so based on these further results, we can hypothesize that the changes in these amino acid levels in saliva may reflect the changes in blood. Additionally, males exhibited significant differences in amino acids levels compared with females, again suggesting differences in amino acids’ utilization.

The main limitation of the study is the number of participants, so it should be considered a pilot study. Furthermore, the findings reported relate to basketball, which is characterized by specific metabolic demands and cannot be generalized to all other sports. Future studies should include a larger number of participants practicing different sports. Finally, we did not consider the menstrual phase of the participants. There are controversial data on the influence of the menstrual cycle on physical activity [56], and it is very difficult to evaluate this.

## 5. Conclusions

In this study, we highlighted that saliva is a useful biofluid to evaluate the performance of athletes, because we found similar results to our previous work involving a plasma analysis of the same subjects [16]. Saliva reflects the cues found in plasma, so it can be used as a valid tool to monitor training and investigate differences between the sexes. 

Regarding adaptation to training, we confirm that males and females share some common characteristics, but also some different sex-specific behaviors, including dissimilar amino acid utilization. This aspect has often been overlooked because women are still underrepresented in sports medicine research compared to men, and further studies should be conducted to fully understand female physiology and metabolism during exercise. Women and men share many gender-specific anthropometric and physiological characteristics that may affect adaptive mechanisms during exercise, and future research should focus on uncovering these differences to allow for more targeted interventions to maintain health, fitness, and performance and treat diseases from a gender perspective. In the future, it may be interesting to examine the effects of different sports that require different levels of effort and types of exercise on the gender differences found in this study. 

## Figures and Tables

**Table 1:** Participants’ characteristics.

Characteristics	Mean (SD)				Tukey’s Test ^a^			
	BM	CM	BF	CF	BM vs. CM	BF vs. CF	BM vs. BF	CM vs. CF
Age (year)	21 ± 2.2	26.1 ± 4.1	25.1 ± 5.5	26.9 ± 2.2	0.01 *	0.77	0.08	0.97
Weight (kg)	81.5 ± 10.2	73 ± 8.7	68.7 ± 11.9	58.7 ± 5.8	0.17	0.17	0.03 *	0.02 *
Height (cm)	186 ± 0.06	178.7 ± 0.06	175.6 ± 0.08	163.4 ± 0.06	0.06	0.003 **	0.006 **	<0.0001 ****
BMI (kg/m^2^)	23.6 ± 2.7	22.9 ± 2.9	22.1 ± 2.05	22 ± 2.3	0.64	1	0.18	0.72

^a^ Tukey’s test was performed by GraphPad Prism 8.0 software between male basket group (BM), male control group (CM), female basket group (BF), and female control group (CF): (* *p*-value < 0.05), (** *p*-value < 0.01), and (**** *p*-value < 0.0001).

**Table 3:** Dermcidin validation by mass spectrometry.

Spot No. ^a^	Accession No. ^b^	Description	Coverage (%) ^c^	Unique Peptides ^d^	Score Mascot ^e^	Score Sequest HT ^f^
31–32	P81605	Dermcidin	21	2	64	2.22
37	P81605	Dermcidin	21	2	70	5.09
43	P81605	Dermcidin	25	4	147	14.15

^a^ Spot numbers reported in the 2DE Western blot shown in Figure 3d. ^b^ Accession number in Swiss-Prot/UniprotKB. ^c^ Percentage of the protein sequence covered by the peptides. ^d^ Total number of peptide sequences unique to the protein group. ^e^ Cumulative protein score based on summing the ion scores of the unique peptides identified. ^f^ Sum of the scores of the individual peptides from the Sequest HT search.

**Table 4:** Differentially expressed metabolites identified in saliva samples by gas chromatography–mass spectrometry (GC–MS) analysis.

Compound Name	CAS Number *	Formula	KEGG ID °	Fold Change/Tukey’s Test			
				BM vs. CM	BF vs. CF	BM vs. BF	CM vs. CF
D-allose	579-36-2	C_6_H_12_O_6_	C01487	−3.1/***	−1.8/*	ns	ns
D-mannitol	87-78-5	C_6_H_12_O_6_	C00392	ns	2.4/***	−2.1/***	ns
L-glutamic acid	56-86-0	C_5_H_9_NO_4_	C00025	3.2/****	2.2/***	ns	ns
L-lysine	56-87-1	C_6_H_14_N_2_O_2_	C00047	5.0/****	3.0/*	ns	ns
L-ornithine	70-26-8	C_5_H_12_N_2_O_2_	C00077	5.2/**	4.0/*	ns	ns
L-threonine	72-19-5	C_4_H_9_NO_3_	C00188	8.9/**	ns	ns	ns
L-valine	72-18-4	C_5_HNO_2_	C00183	5.0/*	ns	ns	ns
O-phosphocolamine	1071-23-4	C_2_H_8_NO_4_P	C00346	−2.2/****	−2.3/**	ns	ns
Citric acid	5949-29-1	C_6_H_8_O_7_	C00158	ns	−1.8/**	−2.5/*	−2.7/***
Glycine	56-40-6	C_2_H_5_NO_2_	C00037	4.9/*	2.5/*	ns	ns
Methyl-beta-D-galactopyranoside	1824-94-8	C_7_H_14_O_6_	C03619	ns	−5.8/****	ns	−8.2/****
N-acetylneuraminic acid	131-48-6	C_11_H_19_NO_9_	C00270	2.8/*	2.0/*	ns	ns
Taurine	107-35-7	C_2_H_7_NO_3_S	C00245	ns	ns	2.6/*	ns
Tyrosine	60-18-4	C_9_H_11_NO_3_	C00082	2.3/**	1.6/*	ns	ns

* Chemical Abstract Service number. ° KEGG identifier (https://www.genome.jp/kegg/). Tukey’s post hoc test was performed on ANOVA *p*-values by GraphPad Prism 6.0 software: (* *p* < 0.05), (** *p* < 0.01), (*** *p* < 0.001), (**** *p* < 0.0001), and (ns = not significant). Fold change was calculated by GraphPad Prism 6.0 software and was reported only for statistically significant values.

## Data Availability

The data presented in this study are available upon request from the authors.

## Supplementary Materials

The following supporting information can be downloaded at https://www.mdpi.com/article/10.3390/healthcare11020241/s1. Table S1: Quantitative data and statistical analyses of protein spots whose intensity levels significantly differed among saliva of the four groups. Table S2: List of Metabolites identified in saliva samples of the subject utilized in this study by gas chromatography–mass spectrometry (GC–MS) analysis.
