# Supplementary material for: Chronic Training Induces Metabolic and Proteomic Response in Male and Female Basketball Players: Salivary Modifications during In-Season Training Programs
Source: Healthcare (Basel). 2023 Jan 12;11(2):241. doi: 10.3390/healthcare11020241 (PMC9858989; doi:10.3390/healthcare11020241)
Supplement: Supplementary file 1 [file healthcare-11-00241-s001.zip › healthcare-2111684-supplementary.pdf]

Supplementary Table S1. Quantitative data and statistical analyses of protein spots whose intensity levels significantly differed among saliva of the four groups.

Spots	Normalized volume (arbitrary unit)[a] Mean (SD)				ANOVA *p*-value[b]	Tukey's test[c]/Fold Change[d]			
	Basket Male	Control Male	Basket Female	Control Female		BM *vs* CM	BF *vs* CF	BM *vs* BF	CM vs CF
1	58.8 ± 24.3	51.6 ± 17.4	27.4 ± 8.8	72.3 ± 19.9	0.036	ns	*	ns	ns
2	35.1 ± 16.6	31.8 ± 3.7	22.7 ± 10.8	59.9 ± 13.5	0.037	ns	*	ns	ns
3	7.5 ± 2.3	21.9 ± 4.5	14 ± 7.4	36.6 ± 5.2	0.001	*	**	ns	*
4	8.4 ± 2.1	17 ± 6.4	9 ± 4.7	30 ± 6.3	0.006	ns	**	ns	ns
5	39.9 ± 1.6	27.6 ± 1.1	21.1 ± 3.8	31.6 ± 2.3	0.0003	**	**	****	ns
6	10 ± 4.2	12.8 ± 3.5	8.9 ± 3.1	21.1 ± 4.5	0.040	ns	*	ns	ns
7	9.6 ± 3.2	18.6 ± 7.1	6.9 ± 1.9	23.5 ± 9.5	0.009	ns	*	ns	ns
8	5.1 ± 1.8	3.3 ± 0.7	2 ± 0.6	6.9 ± 3.1	0.026	ns	*	ns	ns
9	2.9 ± 0.9	6.3 ± 1.7	2.8 ± 2.5	9.1 ± 3.4	0.049	ns	*	ns	ns
10	3.1 ± 1.1	7.1 ± 1.2	4.4 ± 2.3	8.8 ± 2.1	0.018	*	ns	ns	ns
11	2.4 ± 1	1.5 ± 0.4	1 ± 0.3	3.8 ± 1.1	0.007	ns	**	ns	*
12	1.5 ± 0.6	1.6 ± 1	0.4 ± 0.1	2.4 ± 1	0.009	ns	*	ns	ns
13	2.2 ± 0.9	4.1 ± 0.9	4.7 ± 1.1	3.1 ± 1.1	0.061	ns	ns	*	ns
14	3.3 ± 2	6.8 ± 0.5	3.2 ± 1.5	9 ± 0.9	0.020	*	**	ns	ns
15	3.7 ± 2.2	6.4 ± 1.7	2.9 ± 1.4	7.9 ± 1.9	0.033	ns	*	ns	ns
16	1.8 ± 0.2	1.5 ± 1.1	1 ± 0.2	3.1 ± 0.7	0.023	ns	*	ns	ns
17	1.8 ± 0.5	2.6 ± 0.7	3.9 ± 0.6	3.4 ± 0.9	0.023	ns	ns	*	ns
18	0.4 ± 0.1	0.4 ± 0.1	0.2 ± 0.1	1.2 ± 0.7	0.003	ns	*	ns	ns
19	9.2 ± 3.2	13.1 ± 4.9	14.5 ± 5	28.9 ± 1.9	0.016	ns	**	ns	**
20	0.4 ± 0.2	0.4 ± 0.1	0.3 ± 0.02	0.9 ± 0.2	0.005	ns	**	ns	*
21	4.9 ± 1.1	9.5 ± 1.8	7.6 ± 2.9	5.4 ± 0.6	0.035	*	ns	ns	ns
22	11.7 ± 1.2	13 ± 2	9.4 ± 0.9	14 ± 2	0.031	ns	*	ns	ns
23	2.3 ± 1.1	2.3 ± 0.6	1.5 ± 0.8	4.2 ± 0.9	0.044	ns	*	ns	ns
24	0.7 ± 0.2	0.6 ± 0.2	1.9 ± 1	0.5 ± 0.2	0.038	ns	*	ns	ns
25	10.7 ± 5.1	4 ± 1.7	2.4 ± 0.3	6.3 ± 1.5	0.014	ns	ns	*	ns
26	7.1 ± 1.9	3.7 ± 0.9	3.6 ± 0.1	2.2 ± 1.3	0.025	*	ns	*	ns
27	2.7 ± 1.3	4.3 ± 1.1	1.3 ± 0.8	4.5 ± 1.1	0.024	ns	*	ns	ns
28	19.4 ± 7	24 ± 9.6	7.8 ± 1.1	14.1 ± 6.7	0.029	ns	ns	*	ns
29	0.6 ± 0.1	0.2 ± 0.03	0.5 ± 0.2	0.4 ± 0.1	0.007	*	ns	ns	ns
30	10.2 ± 2.5	12.7 ± 2	6.4 ± 2.7	6 ± 1.6	0.041	ns	ns	ns	*
31	34.2 ± 3.6	24.8 ± 8.1	16.8 ± 14.1	6.5 ± 2	0.013	ns	ns	ns	*
32	53.9 ± 21.7	59.2 ± 24.2	22.5 ± 15.4	13.6 ± 4.4	0.034	ns	ns	ns	*
33	10.1 ± 1.7	7.2 ± 1.8	3.7 ± 1.1	3.7 ± 1.8	0.014	ns	ns	**	ns
34	2.4 ± 1	2.6 ± 0.5	1.2 ± 0.2	1.1 ± 0.4	0.026	ns	ns	ns	*
35	1.1 ± 0.3	0.5 ± 0.1	0.3 ± 0.01	0.4 ± 0.3	0.015	*	ns	*	ns
36	29 ± 3.8	34.6 ± 3.3	15.8 ± 1.5	15.1 ± 0.9	<0.0001	ns	ns	**	****
37	12.5 ± 3.3	16.8 ± 2.8	41.5 ± 16.5	7.8 ± 3.8	0.011	ns	*	*	ns
38	3.2 ± 1.7	0.5 ± 0.1	1.5 ± 1.5	0.8 ± 0.1	0.018	*	ns	ns	ns
39	2 ± 0.6	0.3 ± 0.2	0.6 ± 0.2	0.3 ± 0.2	0.022	**	ns	**	ns
40	6 ± 2.8	5.2 ± 1	3.5 ± 1.1	2 ± 0.4	0.026	ns	ns	ns	**
41	50.1 ± 12.2	46 ± 6.9	32.1 ± 7.6	18.5 ± 12.3	0.026	ns	ns	ns	*
42	38 ± 10.5	44 ± 11	31.4 ± 6	20.5 ± 2.2	0.012	ns	ns	ns	*
43	8.5 ± 3	6 ± 1.6	3.3 ± 2	2 ± 1.4	0.034	ns	ns	ns	*

[a] The mean of normalized spot volume and the respective SD were calculated by GraphPad Prism 6.0 software using the normalized volume data calculated by Progenesis SameSpots 4.0 software. All data were reported in order of magnitude 10^{-6}.

[b] ANOVA test was performed by Progenesis SameSpots 4.0 software to determine if the relative change was statistically significant (p<0.05).

[c] Tukey's post-hoc test was performed on ANOVA *p*-values by GraphPad Prism 6.0 software (**p*<0.05), (***p*<0.01), (****p*<0.001), (*****p*<0.0001), (ns= not significant).

[d] Fold change was calculated by GraphPad Prism 6.0 software. It is the ratio of the mean normalized spot volumes of male basket group (BM), male control group (CM), female basket group (BF) and female control group (CF). It was reported only for statistically significant values.

Supplementary Table S2. List of Metabolites identified in saliva samples of the subject utilized in this study by Gas Chromatography–mass spectrometry (GC-MS) analysis.

Compound name	Retention time	Fiehn library number	CAS number*	KEGG ID°
1 monostearin	24.912	24699	123-94-4	
1,3-propanediol	6.777	10442	504-63-2	C02457
1,5-anhydro-D-sorbitol	16.967	219984	154-58-5	C07326
5-aminovaleric acid	14.458	138	660-88-8	C00431
D (+)altrose	17.460	441032	1990-29-0	C06464
D-allose	17.278	448388	579-36-2	C01487
D-mannitol	17.810	6251	87-78-5	C00392
D-sphingosine	22.527	5280335	123-78-4	C00319
L-(+) lactic acid	6.851	107689	79-33-4	C00186
L-glutamic acid	13.232	33032	56-86-0	C00025
L-glutamine	14.083	738	56-85-9	C00064
L-lysine	17.643	5962	56-87-1	C00047
L-norleucine	10.607	21236	327-57-1	C01933
L-ornithine	16.632	6262	70-26-8	C00077
L-proline	10.321	145742	147-85-3	C00148
L-serine	11.174	5951	56-45-1	C00065
L-threonine	11.464	6288	72-19-5	C00188
L-valine	9.151	6287	72-18-4	C00183
N-acetyl-D-mannosamine	19.339	899	7772-94-3	C00645
O-phosphocolamine	16.232	1015	1071-23-4	C00346
Acetohydroxamic acid	7.720	1990	546-88-3	C06808
Beta-gentiobiose	25.420	441422	5996-00-9	C08240
Citramalic acid	12.630	439766	2306-22-1	C00815
Citric acid	16.615	311	5949-29-1	C00158
Eicosapentaenoic acid	24.013	446284	10417-94-4	C06428
Ethanolamine	9.879	700	141-43-5	C00189
Galactinol	26.479	439451	3687-64-7	C01235
Glycerol 1-phosphate	16.056	754	34363-28-5	C03189
Glycine	10.456	750	56-40-6	C00037
Hydrocinnamic acid	11.900	107	501-52-0	C05629
Malonic acid	8.919	867	141-82-2	C00383
Maltitol	25.370	16217663	585-88-6	C05399
Methyl-beta-D-galactopyranoside	16.935	94214	1824-94-8	C03619
N-acetylneuraminic acid	23.855	445063	131-48-6	C00270
Oxalic acid	7.883	971	144-62-7	C00209
Palmitic acid	18.846	985	64519-82-0	C00249
Phosphoric acid	9.966	1004	7664-38-2	C00009
Pipecolic acid	11.220	439227	7664-38-2	C00009
Psicose	17.059	90008	551-68-8	C06468
Rhamnose	15.574	220001	3615-41-6	C00507
Ribitol	15.660	827	488-81-3	C00474
Succinic acid	10.509	1110	29915-38-6	C00042

Taurine	14.940	1123	107-35-7	C00245
Tyrosine	17.871	6057	60-18-4	C00082
Urea	9.599	1176	57-13-6	C00086
Uric acid	19.331	1175	66-22-8	C00366
Xylitol	15.376	6912	87-99-0	C00379
Xylose	14.700	6027	58-86-6	C00181

* Chemical Abstract Service number; ° KEGG identifier (https://www.genome.jp/kegg/).
